# Toward a Digital Platform for the Self-Management of Noncommunicable Disease: Systematic Review of Platform-Like Interventions

**DOI:** 10.2196/16774

**Published:** 2020-10-28

**Authors:** Sarah A Tighe, Kylie Ball, Finn Kensing, Lars Kayser, Jonathan C Rawstorn, Ralph Maddison

**Affiliations:** 1 Institute for Physical Activity and Nutrition (IPAN) School of Exercise and Nutrition Sciences Deakin University Burwood Australia; 2 Department of Public Health Faculty of Health and Medical Sciences University of Copenhagen Copenhagen Denmark; 3 Department of Computer Science Faculty of Science University of Copenhagen Copenhagen Denmark

**Keywords:** noncommunicable diseases, chronic disease, web-based intervention, mobile health, self-management, health behavior, mobile phone

## Abstract

**Background:**

Digital interventions are effective for health behavior change, as they enable the self-management of chronic, noncommunicable diseases (NCDs). However, they often fail to facilitate the specific or current needs and preferences of the individual. A proposed alternative is a digital platform that hosts a suite of discrete, already existing digital health interventions. A platform architecture would allow users to explore a range of evidence-based solutions over time to optimize their self-management and health behavior change.

**Objective:**

This review aims to identify digital platform-like interventions and examine their potential for supporting self-management of NCDs and health behavior change.

**Methods:**

A literature search was conducted in January 2020 using EBSCOhost, PubMed, Scopus, and EMBASE. No digital platforms were identified, so criteria were broadened to include digital platform-like interventions. Eligible platform-like interventions offered a suite of discrete, evidence-based health behavior change features to optimize self-management of NCDs in an adult population and provided digitally supported guidance for the user toward the features best suited to their needs and preferences. Data collected on interventions were guided by the CONSORT-EHEALTH (Consolidated Standards of Reporting Trials of Electronic and Mobile Health Applications and Online Telehealth) checklist, including evaluation data on effectiveness and process outcomes. The quality of the included literature was assessed using the Mixed Methods Appraisal Tool.

**Results:**

A total of 7 studies were included for review. Targeted NCDs included cardiovascular diseases (CVD; n=3), diabetes (n=3), and chronic obstructive pulmonary disease (n=1). The mean adherence (based on the number of follow-up responders) was 69% (SD 20%). Of the 7 studies, 4 with the highest adherence rates (80%) were also guided by behavior change theories and took an iterative, user-centered approach to development, optimizing intervention relevance. All 7 interventions presented algorithm-supported user guidance tools, including electronic decision support, smart features that interact with patterns of use, and behavior change stage-matching tools. Of the 7 studies, 6 assessed changes in behavior. Significant effects in moderate-to-vigorous physical activity were reported, but for no other specific health behaviors. However, positive behavior change was observed in studies that focused on comprehensive behavior change measures, such as self-care and self-management, each of which addresses several key lifestyle risk factors (eg, medication adherence). No significant difference was found for psychosocial outcomes (eg, quality of life). Significant changes in clinical outcomes were predominately related to disease-specific, multifaceted measures such as clinical disease control and cardiovascular risk score.

**Conclusions:**

Iterative, user-centered development of digital platform structures could optimize user engagement with self-management support through existing, evidence-based digital interventions. Offering a palette of interventions with an appropriate degree of guidance has the potential to facilitate disease-specific health behavior change and effective self-management among a myriad of users, conditions, or stages of care.

## Introduction

### Background

Noncommunicable diseases (NCDs) are the leading cause of death and disability worldwide [1,2]. Although complex [3-5], approximately 80% of NCDs can be accounted for by modifiable risk behaviors, such as physical inactivity, unhealthy diets, smoking, harmful alcohol consumption, and stress management [3,6,7]. The comprehensive management of risk behaviors [8,9] through the implementation of self-management strategies [10-12] is critical to fostering a life-long approach to secondary prevention [13]. According to Bandura [14], to achieve successful and sustainable self-management, *people have to learn to monitor their health behavior and the circumstances under which it occurs*.

Secondary prevention in an NCD context usually involves referral to a structured hospital or community-based programs, such as cardiac rehabilitation [13,15,16], pulmonary rehabilitation [17], or diabetes education [18-21]. Participation in center-based programs improves health-related quality of life [12,22-24] and clinical outcomes [23] and lowers the risk of hospitalization [12,22,24], recurring adverse health-related events, and all-cause mortality [9], compared with usual care.

Despite the proven effectiveness of secondary prevention [12,22-24], uptake and adherence to structured face-to-face programs is suboptimal [25-29]. Personal factors associated with low attendance include cultural, financial, and psychological barriers (eg, readiness or willingness to attend) [17,27,30]. Key operational barriers to active participation relate to availability of program resources, including limited program enrollment capacity [29,31], restrictive hours of operation [27], and limitations in program suitability (eg, for people self-managing multiple NCDs). Accessibility barriers include inadequate transportation and a lack of services within remote rural areas [17,27,29,30,32-36]. In response, there has been a necessary shift toward flexible and convenient home-based services [32,37-41], offering an evidence-based alternative to nonattendance and reducing the distance of care [42].

Digital health is a contemporary advancement in home-based self-management of NCDs. Widespread internet connectivity to at least 55% of the global population [43], over 5 billion mobile phone users [44] and the availability of thousands of mobile health applications [45] has provided unprecedented access to the digital delivery of home-based support. Content, structures, and modes of delivery for these digital interventions are wide ranging, and their potential effects are well documented.

A systematic review and meta-analysis of internet-based interventions targeting health behavior change in NCD groups (n=43,236) showed significant improvements in risk-related behavior, such as physical activity (effect size 0.24; 95% CI 0.09-0.38), dietary behavior (effect size 0.20; 95% CI 0.02-0.37), and alcohol consumption (effect size 0.14; 95% CI 0.00-0.27) [46]. The findings showed that offering multiple modes of delivery within an intervention (eg, internet-based plus text messaging) had a greater effect on behavior (effect size 0.81; 95% CI 0.14-1.49) [46].

Reviews of communication technologies used to deliver health services and facilitate patient and health care professional interaction [47] have reported that improvements in self-management of NCDs are not inferior to the positive changes produced by structured center-based programs or usual care [42,48]. Similarly, reviews of mobile-based text messaging interventions have shown sustained health behavior change [49-53] and the potential to overcome barriers associated with traditional center-based models via simple to use, flexible, cost-effective digital health [52,53].

Collectively, the literature indicates that evidence-based digital health interventions have the potential to discretely impact the self-management of NCDs while also complementing one another. Despite the advantages of these discrete interventions, no single program meets the needs of all users, as self-management of NCDs and user preferences are complex and multifaceted in nature. Discrete digital health solutions could be complimentary when combined for use. The REMOTE-CR program [54] and CAP-CR program [55] draw on combinations of web-based and mobile-based features through smartphone-enabled software to facilitate digitally assisted self-management. Outcomes from a randomized control trial of the CAP-CR intervention (n=120) indicated that uptake, adherence, and completion rates at 6 months were significantly higher than those of center-based cardiac rehabilitation programs [55]. REMOTE-CR integrates the use of smartphones, wearable sensors, and web apps to provide real-time remote exercise monitoring. A randomized control trial (n=162) found REMOTE-CR to be an effective and cost-efficient alternative to center-based programs [54].

The constraints of disparate digital health interventions mean that an undue amount of time is required to seek out interventions offering the content, features, or delivery mode best suited to the current preferences of the individual user. Moreover, when a person’s health status or general lifestyle habits change, seeking practices would have to be repeated, which may reduce the sustainability of a discrete digital program intervention and long-term user adherence [56].

Therefore, we need flexible and versatile solutions in the digital health space. This requires a shift *away* from the creation of restrictive digital interventions *toward* a paradigm that facilitates optimal engagement through centralized choice.

### Objective

We propose a digital platform that would capitalize on existing digital self-management interventions that have been evaluated for effectiveness in a specific context (eg, NCDs) through a comprehensive experimental design [57]. The platform would host a digitally supported palette of discrete, evidence-based digital health interventions and incorporate a digital guidance tool to direct users to the intervention best suited to their current individual needs and preferences, optimizing personal relevance and user experience [47].

Existing disparate digital health interventions may offer modest positive effects, but engagement can be varied. Thus, a digital platform could potentially optimize engagement while also lessening the burden of care associated with irrelevant content, user ambivalence, and time-consuming seeking processes. Such an approach not only facilitates personalization but also encourages user autonomy through the self-selection of a combination of program components, which is associated with long-lasting positive effects on disease management and patient empowerment [37,58,59]. There is significant potential for this body of work, as a plethora of underutilized evidence-based digital health interventions exists, involving rigorous development processes and gold standard evaluations.

## Methods

### Identifying Digital Platforms

A preliminary pilot of this review was conducted in August 2018, but no digital platforms that matched our criteria were identified. Therefore, the focus of this review was extended to include digital *platform-like* interventions. Details of the protocol for this review were registered on PROSPERO (International Prospective Register of Systematic Reviews) in 2018 (PROSPERO 2018, Registration Number: CRD420 18102095). A follow-up literature search was conducted in January 2020.

For this review, a digital platform-like intervention was defined as *a digital solution that allows users to choose from a suite of discrete, evidence-based health behavior change features to support NCD self-management. Offers a digital tool for guidance toward intervention features that are most suited to the user’s needs and preferences*. This broadened definition allowed us to best provide an assessment of the potential of digital platforms in self-management of NCDs, despite the absence of existing literature.

### Aims

The aims of this study were to identify digital platform-like interventions for the self-management of NCDs and to examine the potential for digital platform-like interventions to support self-management of NCDs through effectiveness and process outcome measures.

### Eligibility Criteria

The eligibility criteria are outlined in Table 1 according to an adapted version of the *P*opulation, *I*ntervention, *Co*ntext, *O*utcome approach [60,61]. Studies eligible for inclusion were published in English between January 1990 and January 2020; this period coincides with the activation of commercial internet service providers. All study designs were eligible for inclusion because of the emergent nature of the research topic.

**Table 1:** Framing the review aims according to an adapted Population, Intervention, Context, Outcome approach.

Factor		Description	
**Population**			
	Adults		Aged ≥18 years
	Noncommunicable chronic disease		Intervention supported participants in self-management of at least one lifestyle-related, noncommunicable, chronic disease (eg, cardiovascular disease, chronic obstructive pulmonary disease, and type 2 diabetes)
**Intervention**			
	Health behavior		Intervention targeted at least one health behavior (eg, physical activity or diet)
	Discrete behavior change components		Offered at least two evidence-based BC^a^ components or features (eg, self-monitoring, goal setting, or feedback)
	Digital delivery		Compatible with any modern computing devices (eg, web based or mobile based)
	Guidance		Offered a choice between BC components. Provided users with a digitally assisted guidance tool to assist with feature selection.
**Context**			
	Secondary prevention and self-management		Primarily facilitating outpatient, home-based stages of NCD^b^ care. Focus on participants’ own self-management of NCD.
**Outcomes**			
	Intervention description		Mode of delivery, participant information, comparators, intervention features and components, theoretical frameworks or tools, development processes.
	Effectiveness		Significant improvements in health behavior, clinical outcomes, or evidence-based psychosocial outcomes.
	Process		Intervention use (log-in data), adherence (completion of follow-up data collection), and user satisfaction (quantitative or qualitative follow-up).

^a^BC: behavior change.

^b^NCD: noncommunicable disease.

### Information Sources and Search Strategy

A systematic literature search of 9 electronic databases was conducted in January 2020: EBSCOhost (Academic Search Complete, Applied Science & Technology Source, Cumulative Index to Nursing and Allied Health Literature Complete, Global Health, Health Business Elite, and PsycINFO), PubMed (MEDLINE), Scopus, and EMBASE. Reference lists of included study publications and related conference proceedings were hand searched to identify additional publications that may not have appeared in database searches. Peer-reviewed systematic reviews and meta-analyses, in which the included studies were cited, were explored to identify any similar studies that did not appear in database searches.

Individual search strategies were developed for each database (example shown in Multimedia Appendix 1) and included search terms derived from 3 main categories of interest: chronic disease, digital technology, and self-management of health behavior.

### Screening and Selection

**Figure 1:**
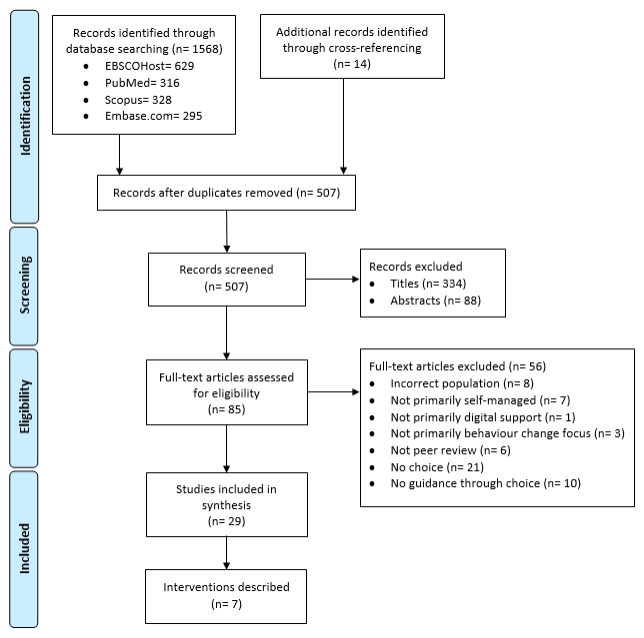
PRISMA (Preferred Reporting Items for Systematic Reviews and Meta-Analyses) flowchart.

All results were exported to a reference manager (EndNote X9, Clarivate Analytics), where duplicates were removed before screening for eligibility. Search results have been reported using the PRISMA (Preferred Reporting Items for Systematic Reviews and Meta-Analyses) statement [62]. Each stage of the screening process is presented in Figure 1. Titles, abstracts, and full texts were independently assessed by 2 reviewers (ST and JR), whereas the third and fourth reviewers (KB and RM) were consulted to collectively reach a consensus on studies with questionable eligibility.

### Data Collection

#### Quality

Methodological quality of the included literature was assessed using the Mixed Methods Appraisal Tool (MMAT; version 18) [63]. The MMAT allows for the simultaneous evaluation of qualitative, quantitative, and mixed-method research, which makes it an appropriate critical appraisal tool for this review.

Data were extracted from the publications using a specifically designed MMAT tool. Publications were not excluded based on quality assessment because of little empirical evidence to support this practice [63]. Instead, we decided to report on the quality of the reviewed studies.

The quality of each eligible study was rated according to 7 quality criteria. To determine the overall quality score for each study, the number of criteria met was divided by the total number of criteria (7) and expressed as a percentage. It is discouraged to simply calculate an overall score [63]; thus, a more detailed report of the criteria was included to better inform the quality assessment of the included studies.

#### Digital Platform-Like Interventions

The extraction of intervention data was guided by the CONSORT-EHEALTH (Consolidated Standards of Reporting Trials of Electronic and Mobile Health Applications and Online TeleHealth) checklist [64], which outlines reporting guidelines for digital health interventions. Data on mode of delivery, participant information, comparators, intervention features and components, and theoretical frameworks or tools were collected. Data were also collected on the development processes of the digital platforms.

#### Evaluation

According to highly regarded reports on monitoring and evaluation frameworks for digital health interventions [57,65-67], summative and process evaluations are typically carried out in line with the stage of maturity or specific characterization of a digital platform.

For this review, impact measures for effectiveness were classified as significant improvements in health behavior, clinical outcomes, or evidence-based psychosocial outcomes. Outcome measures for process evaluations were intervention use [68] (log-in data), adherence [68] (completion of follow-up data collection), and user satisfaction (quantitative or qualitative follow-up).

## Results

### Study Selection

The combined search strategy identified 1582 records, of which 1568 were identified through electronic database searching and 14 through other sources. Duplicates were removed, and the remaining 507 records were screened for eligibility through titles and abstracts. Full-text was obtained for the 85 remaining records, of which 29 publications outlining 7 digital platform-like interventions met the inclusion criteria (Table 2) [38,69-95]. The primary reasons for the exclusion of full-text records are shown in Figure 1. Table 2 shows how the 29 publications were related to each of the 7 studies, sorted according to the evaluation stage [57]. Owing to the heterogeneous nature of the included studies, a meta-analysis was not possible.

**Table 2:** Overview of included publications (n=29).

Study name	Proposal or protocol (n=7)		Formative evaluation		Summative evaluation							Formative evaluation	
	Year	Reference	Needs assess		Iterative development				Trial		Process evaluation		
			Qualitative (n=4)		Usability or feasibility (n=5)		Pilot or efficacy (n=5)		Effect (n=4)		Mixed methods (n=4)		
			Year	Reference	Year	Reference	Year	Reference	Year	Reference	Year		Reference
Antypas and Wangberg [71]	2012	[69]	2014a	[70]	—^a^	—	—	—	2014b	[71]^b^	—		—
Murray et al [88]	2015	[96]	2018 (Pal)	[38]	2019 (Dack)	[91]	2016 (Hofmann)	[86]	2017	[88]^b^	2017 (Alkaldi)		[87]
	—	—	—	—	—	—	—	—	—	—	2018 (Li)		[89]
	—	—	—	—	—	—	—	—	—	—	2018 (Poduval)		[90]
Poppe et al [93]	2019a	[92]	2017	[72]	2018	[73]	2019b	[93]^b^	—	—	—		—
Sakakibara et al [94]	2019	[95]	—	—	—	—	2017	[94]^b^	—	—	—		—
Voncken-Brewster et al [77]	2013a	[74]	—	—	2013b	[75]	2014	[76]	2015	[77]^b^	2017		[78]
Walsh et al [82]	2017 (Claes)	[79]	2018	[80]	2019	[81]	2020 (Claes)	[82]^b^	—	—	—		—
Yu et al [85]	2012	[83]	—	—	2014a	[84]	—	—	2014b	[85]^b^	—		—

^a^Publication type not applicable to this study

^b^Parent publication for the study.

### Study Characteristics

This review identified 7 diverse digital platform-like interventions [38,69-95]. Of the 7 interventions, 3 targeted diabetes [85,88,93], 3 were intended for cardiovascular diseases (CVDs) [71,82,94], and only one intervention supported people with chronic obstructive pulmonary disease (COPD) [77].

Of the 7 studies, 4 evaluated the effectiveness of their interventions [71,77,85,88], including 3 randomized control trials and 1 single-arm pre-post cohort design [85]. Sample sizes ranged from 69 to 1325, and mean participant ages ranged from 57 to 65 years. Of the 4 studies, 2 reported gender imbalance within participant groups (23% [71] and 31% [88] female participants). Trial periods ranged from 3 to 12 months, and the control groups in the 3 randomized control designs received the usual care [77], accessed simple web-based information [88], or a variation of the described intervention [71].

Of the 7 studies, 3 had not evaluated effectiveness at the time of this review [82,93,94], but all 3 had completed a pilot evaluation of their intervention. Pilot sample sizes ranged from 35 to 120 and had relatively shorter trial periods of 5 weeks [93], 10 weeks [94], and 6 months [82]. One of the studies subsequently published a protocol for a planned randomized control trial [95] to be completed by December 2020 (clinical trial registration: NCT03159325; international registered report identifier: DERR1-10.2196/12322).

### Quality Appraisal

Parent publications for each study [71,77,82,85,88,93,94] were quality assessed; thus, the MMAT was completed once per study and not for each publication. Companion publications were assessed where a *Can’t Tell* response was coded [38,69-96]. Overall, 6 of the digital platform-like interventions were intended to be used ad libitum. Therefore, the criterion “2.5. Did the participants adhere to the assigned intervention?” was not appropriate. This section was alternatively appraised based on the participants’ completion of follow-up data collection.

The details of the assessment criteria can be found in Multimedia Appendix 2 [71,77,82,85,88,93,94]. Overall, the scores for the included studies ranged from 3 to 7 out of a possible maximum of 7. This translates to a varied methodological quality range of 2 to 4 stars. The mixed-method studies were of the highest quality, achieving 3 [94] and 4 stars [85]. The most frequent limitations were incomplete outcome data sets [71,77,82,88,93], poor follow-up response rates, [71,77,82,88,93], and insufficient integration of quantitative and qualitative components in mixed-method studies [94].

### Digital Platform-Like Interventions

The key characteristics of the included interventions are outlined in Table 3. All interventions included an intermediary user interface, which allowed participants to explore and self-select optional components based on their individual needs and preferences. All 7 delivered a choice of at least two evidence-based behavior change techniques or strategies (eg, self-monitoring and goal setting) [97] targeting a lifestyle-related behavior (eg, physical inactivity and smoking). Physical activity was the most frequently targeted health behavior, addressed in 6 of the 7 digital health interventions [71,77,82,85,88,93]. Of the 7 interventions, 2 focused on disease-specific self-care as their key targeted behavior, which incorporates a wide range of health behaviors [85,94].

All 7 interventions presented algorithm-supported interventions that facilitated user guidance [71,77,82,85,88,93,94]. Specific features were included to optimize the sustainability of this automated approach, such as providing recommendations to users through computer-generated responses to questionnaires or self-reported data (ie, eDecision support tools) [73,77,82,88,93,94], smart recommendation widgets that offered advice based on individual patterns of use [85], and application of the transtheoretical model (TTM) of behavior change to select stage-matched intervention features [71].

Support from a health care professional was described in all 7 interventions: typically, cardiac rehabilitation nurse practitioners, exercise specialists, or a member of the research team. The primary role of health care professionals was to facilitate trial and data collection [71,77,82,85,88,93,94] or conduct initial familiarization sessions [71,82,88]. Overall, health care professionals did not provide digital support or enhance existing intervention features, as this approach was not considered sustainable [71,83].

**Table 3:** Overview of digital platform-like interventions.

Intervention name		Country	Mode of delivery	Target NCD^a^	Targeted health behaviors	Intended use
**Antypas and Wangberg [69-71]**						
	Drupal	Norway	Web based and mobile based	CVD^b^	Physical activity, medication adherence, diet/nutrition, and smoking cessation	Ad libitum
**Murray et al [38,86-91,96]**						
	HeLP-Diabetes	England	Web based and mobile based	T2D^c^	Physical activity, medication adherence, diet/nutrition, and smoking cessation	Ad libitum
**Poppe et al [72,73,92,93]**						
	My Plan 2.0	The Netherlands	Web based and mobile based	T2D	Physical activity and sedentary behavior	1 session per week
**Sakakibara et al [94,95]**						
	Healing Circles	Canada	Web based and mobile based	CVD	Multiple, unspecified: related to CVD^a^	Ad libitum
**Voncken-Brewster et al [74-78]**						
	MasterYour Breath	The Netherlands	Web based	Chronic obstructive pulmonary disease	Physical activity, smoking cessation	Ad libitum
**Walsh et al [79-82]**						
	PATHway	Ireland, Belgium	Web based and sensor based	CVD	Physical activity (primary) and other CVD-related	Ad libitum
**Yu et al [83-85]**						
	Diabetes Online Companion	Canada	Web based	T2D	Multiple, unspecified: related to T2D	Ad libitum

^a^NCD: noncommunicable disease

^b^CVD: cardiovascular disease.

^c^T2D: type 2 diabetes.

#### Development Processes

Of the 7 interventions, 6 had a clear theoretical basis to support the concept and development, including behavior change theories such as social cognitive theory (SCT) [81,83,88], social ecological model [88], the Health Action Process Approach (HAPA) [69,73], and the wide-ranging integrated change model (iChange) [69,74]. Of the 7 interventions, 1 was not explicit about the theoretical underpinning but was influenced by aspects of SCT [95]. Of the 7 studies, 3 [82,88,93] outlined platform-like intervention components according to the behavior change technique taxonomy of Michie et al [97].

Of the 7 studies, 4 conducted an early stage needs assessment before commencing development [38,70,72,80] to verify the unmet requirements of their target NCD population [38,70,80] and validate their intervention concept [72]. These needs assessments took the form of focus groups [38,70], semistructured interviews [80], or think-aloud sessions with existing digital infrastructures [72].

Of the 7 digital interventions, 4 took an iterative development approach [75,81,84,91], which involved target participants with NCD throughout the design process of the digital platform-like intervention. This approach included at least three iterative cycles [75,81,84,91], and in some cases, the process was guided by participatory design principles [81,91].

In contrast, just one of the 7 interventions did not involve participants in the development process [94], choosing to focus on key theories and researcher expertise to conceptualize and create the Healing Circles intervention.

### Evaluation

All 7 digital platform-like interventions were evaluated (Multimedia Appendix 3; [71,77,82,85,88,93,94]) through either preliminary pilot investigations [82,93,94] or effectiveness trials [71,77,85,88]. Of the 7 interventions, 2 used evidence-based self-report measures for data collection [71,77]; 2 other studies collected data during researcher visits to participants [82,93] and from hip-worn ActiGraph accelerometers; and 3 collected data through a combination of existing medical records [85,94], self-report measures [85,88,94], and researcher visits to participants [85,88].

#### Behavioral Outcome Measures

Of the 7 studies, 4 reported on physical activity behavior change [71,77,82,93], one of which demonstrated significant improvements in overall physical activity (median 5613 metabolic equivalents of task-minutes [MET-min] per week, IQR 2828) compared with the control group at 3 months (median 1356 MET-min per week, IQR 2937) [71]. For specific intensities, changes in physical activity behavior for the intervention group were significantly better than that for the control for walking only (+453.8 MET-min per week; *P*=.05) [71]. Accelerometer-assessed moderate-to-vigorous physical activity (MVPA) behavior was significantly improved from baseline in 2 of the digital platform-like interventions [82,93]. PATHway reported an increase of approximately 14 min MVPA per day (*P*=.04) at 6 months [82], and the My Plan 2.0 intervention group increased MVPA from baseline by approximately 8 min per day [93]. Both interventions resulted in medium-to large-interaction effects between the intervention and control groups [82,93].

Change in self-care behavior was the primary outcome for one of the studies that targeted diabetes [85]. The Summary of Diabetes Self-Care Activities measure [98] indicated that significant improvements in diabetes self-care behavior and sustained changes (9 months from baseline) were positively correlated with age (+0.04 per year, 95% CI 0.02-0.06; *P*<.001) [85].

Improvements in self-monitoring behavior were observed in 2 of the 7 interventions [93,94]. My Plan 2.0 found a significant time-group intervention effect favoring the intervention group for self-monitoring (effect size 0.54; *P*=.008) [93]. At 10 weeks, the Healing Circles intervention also reported a time effect for the intervention group (z=−2.04; *P*=.04) [94]. The effect of Healing Circles on self-monitoring behavior was emulated by the self-management domains of health behaviors (z=−2.11; *P*=.04) and social support (z=−2.58, *P*=.01), all 3 of which were assessed through the Health Education Impact Questionnaire to measure the changes in self-management behavior [99].

Smoking cessation [77], dietary changes [82], medication adherence [82], alcohol use [82], and stages and mediators of health behavior change [71,77,82] were examined in 3 of the 7 studies, but no significant effects were found.

#### Clinical Outcome Measures

Disease-specific clinical outcomes were reported in 4 studies at 6 months [77,82], 9 months [85], and 12 months [88]. One of the publications demonstrated significant improvements in clinical disease control (−0.06, 95% CI −0.11 to −0.01; *P*=.01) using the 10-item Clinical COPD Questionnaire [100]; however, this effect was not maintained when corrected for participant baseline characteristics, such as age, sex, and disease status [77]. At 6 months, a CVD-focused study [82] reported medium-sized group interactions in favor of the intervention for diastolic blood pressure (effect size=−0.49; *P*=.004) and cardiovascular risk (effect size=−0.36; *P*=.03; Framingham Risk Score [101]). One platform-like intervention for diabetes reported lowered glycated hemoglobin (HbA_1c_) in the intervention group at 12 months compared with the control group who had access to web-based information [88]. Further causal analyses of *high-usage* participants within the intervention group indicated that intervention use for greater than or equal to a median of 4 days could potentially reduce HbA_1c_ levels by 0.44% over 12 months [88]. Another study measured weight, diabetes-related blood markers, and blood pressure, but no overall positive effects were identified [85].

#### Psychosocial Outcome Measures

Health-related quality of life, disease-related quality of life [77,82,85,94], and self-efficacy [71,82,85,88,93] were the most prevalent psychosocial outcomes collected across the 7 studies, but 6 of the 7 reported no significant change. Significant improvements were recorded for disease-related quality of life (using Diabetes Distress Scale [102]) in the diabetes companion intervention group [85] when comparing users (n=70) with nonusers (n=11) at 9 months (−4.7 vs −0.9; *P*<.001).

#### Process Evaluation

Log-in data were measured in 6 of the 7 interventions [71,73,78,82,85,88], and adherence (completion of follow-up data collection) was measured in all 7 interventions [71,77,82,85,88,93,94]. One of the 7 interventions reported that 86% of participants logged in to the intervention at least once over 9 months [85]. Of these participants, 75% were classified as infrequent users (<2 log-ins per month) with an average of one log in per user per month [85] and an average time of 6 min spent per log in. One of the 7 interventions explained that although participant log-in rates averaged 8 sessions per month (range 0.6-3.6 sessions per week) [82], usage dropped significantly in the final 2 months of the 6-month trial (*P*<.001). Another one of the 7 interventions [78] recorded a baseline log-in of 59.5% for the intervention group, which was significantly higher than the initiation rates for the control group program (12.8%). This disparity between the intervention and control groups was also reported in another intervention [88]. Mean log-in values were significantly higher in the intervention group (18.7 vs 4.8; *P*<.001), averaging about 1.5 log-ins per month over the 12-month trial [88]. One of the intervention protocols required users to log in 5 times over 5 weeks [73]. Overall, 92% of the participants logged in at least once, and the average total time spent using the intervention was about 49 min [73]. One of the 7 interventions intended to measure log-in rates [71], but unforeseen technical issues meant that some data were unreliable.

Overall, the mean adherence to intervention trials at follow-up was 69% (SD 20%). In 4 of the 7 interventions, approximately 80% of participants successfully completed follow-up data collection at 6 months [77,82], 9 months [85], and 12 months [88]. For 2 shorter trial periods, over 60% of participants adhered to data collection following a 5-week [93] and 10-week [94] intervention. In contrast, another intervention demonstrated a low responder rate of just 27% 3 months from baseline [71]. Of the 7 studies, 4 used email and SMS reminders to secure follow-up data [71,77,88,94], 3 of which reported adherence rates of over 60% [77,88,94].

Of the 7 interventions, 6 reported on user satisfaction, which was evaluated through both qualitative and quantitative measures 71,73,78,85,88,94

Of the 7 studies, 4 used semistructured exit interviews to gauge user satisfaction [73,78,85,94] and conducted a quantitative follow-up for satisfaction at the end of the trial by asking the user if they would recommend the intervention to a friend [71,78] or by assessing changes in the diabetes treatment satisfaction questionnaire [88]. One of the interventions used a mixed-method approach [78] using both data collection methods.

Participants commented that they were satisfied with the intervention layout, navigation, and ease of use [73,78,85]. The interventions were seen as evidence-based, authoritative sources [78,85], which enhanced users’ accountability for their self-management and behavior change goals [73,85]. One participant group noted that accountability could have been further enhanced by including more social support (eg, intervention access for family members or friends) [73], which was supported by user satisfaction for a social support component of another intervention (peer-to-peer web-based interaction) [85]. The personal relevance of the platform-like interventions was noted as a key contributor to overall user satisfaction [73,78,85], driven by users’ individual contexts and circumstances. Moreover, users were also satisfied with the interventions’ ability to accommodate a broad range of user needs [73,85]. Although users valued the personal relevance of the interventions, there was some dissatisfaction with the burden of a high volume of questions associated with digital guidance tools [73,78]. User dissatisfaction was also reported for interventions that did not offer a mobile delivery option [78,85] and by users who were self-managing more than one chronic condition [78,85]. User satisfaction outcomes were mixed for the 3 quantitative evaluations. Overall, 57% [78] and 68% [71] of participants would reportedly recommend the intervention to a friend, but the HeLP-Diabetes intervention [88] reported no significant difference between intervention and control group satisfaction outcomes.

## Discussion

### Principal Findings

This review is the first of its kind to systematically examine the literature on digital platform-like interventions for the self-management of NCDs and health behavior change. Although no digital platforms were identified, an examination of digital platform-like interventions has contributed knowledge to the conceptualization and potential value of using digital platform architectures to support self-management of NCDs.

A total of 7 digital platform-like interventions were included in the review. Evaluations for effectiveness were disparate, and so a consensus on the overall effect could not be reached. Nevertheless, positive effects were reported for physical activity, disease-specific self-care, and self-monitoring behaviors, which is a promising finding in support of digital platform use for the self-management of NCDs. As a result of the findings mentioned earlier, 3 dominant themes emerged: development, optimizing change, and support and guidance for users.

### Development

This review found that comprehensive and systematic development processes were implemented for most platform-like interventions. Iterative, user-centered approaches are highly regarded in the field of digital health research because the proactive engagement of patients can be beneficial in the development of digital interventions [103,104]. It allows researchers to gain an in-depth understanding of the psychosocial context of the potential end users of the intervention [105] and shortens the communicative distance between the researcher and the user [106]. In this review, 4 of the 7 interventions [75,81,84,91] adopted an iterative, user-centered approach. Reporting methods across the included interventions were varied, so it was difficult to determine whether incorporating user-centered development was associated with greater effectiveness. However, research suggests that such development methodologies have the most potential for developing a sustainable solution [107]. An average of 80% adherence at follow-up stages was found in the same 4 studies [77,82,85,88], which could be attributed to the comprehensive iterative development approaches used in all 4 of the interventions. The level of user engagement with digital health technology tends to wane over time [56,108,109], which has been associated with a lack of perceived value by the user and increased burden because of irrelevant material [110,111]. Placing the user at the center of development considerations and including them in decision-making processes may have affected the creation of fitting platform-like structures, appropriate for those living with NCDs and delivering relevant, usable content or features.

All 4 of the aforementioned interventions [75,81,84,91] also specified a clear theoretical underpinning to their development (eg, TTM), which is consistent with research recommendations that intervention content and features associated with a solid theory base are more likely to be effective in changing behavior [46]. A strong theoretical framework may also have affected the generalizability and adaptability of the platform-like interventions, further adding to the potential for efficacy and longevity [66,112,113] as seen through high adherence rates. Only 3 of the studies outlined platform-like intervention components according to a taxonomy [82,88,93], which made identifying behavior change features difficult because of vague reporting. We anticipate that digital platform content would have superior clarity for users and health care professionals alike because of the stand-alone, discrete digital interventions offered.

### Optimizing Change

The results presented in this review indicate the potential for digital platforms to affect behavior change, such as disease-specific self-care behavior. Significant differences were reported for sustained diabetes self-care behavior between users and nonusers of the Diabetes Online Companion intervention [85].

The self-care outcome measure covers a range of health behaviors, which address several key lifestyle risk factors (eg, dietary behavior, physical activity, glucose monitoring, and smoking) [98]. Thus, our findings suggest that modest changes in multiple health behaviors (ie, changes in self-care) may have a better overall and sustained effect on the self-management of NCDs than larger effects in one single health behavior. This suggestion supports the opinion that affecting comprehensive lifestyle change may be a better approach to the self-management of NCDs, as NCDs are complex conditions influenced by several interconnected lifestyle risk factors [3,114]. Similar to self-care, improvements in self-monitoring behavior were identified through the HAPA, which incorporates six personal determinants of behavior change.

Lifestyle-related risk factors and their corresponding health behaviors are heavily influenced by one another, and thus modifying one health behavior using a digital platform may not necessarily generate sustainable improvements in the self-management of NCDs. For example, smoking behavior has been inversely linked to physical activity behavior, meaning that a lack of engagement with smoking cessation may inhibit physical activity progress [115-117]. Similarly, poor stress management may have a negative effect on engagement with healthy behaviors, such as smoking cessation [118], physical activity [119,120], and maintaining a healthy diet [121]. Single interventions that independently target diet and physical activity behavior may improve those isolated behaviors, but sustained changes in associated risk factors (eg, weight loss and maintenance) are more probable when interventions simultaneously target both health behaviors [114]. The results of our review showed limited to no changes in isolated behaviors (eg, overall physical activity and smoking) [77,82], but modest improvements were apparent in overall disease-specific self-care and multiple self-management domains [85,93,94].

Focusing on modest overall improvements in health behavior change may produce further consequential effects on sustained NCD self-management. Self-regulatory and self-efficacy theories for promoting self-management suggest that the greatest improvements in self-care of chronic conditions typically occur following some initial success in changing behavior (ie, mastery experience) [14]. Thus, seeking a modest change in a more comprehensive and generalizable outcome measure such as disease-specific self-care may generate a cascade of behavior change improvements moving forward.

A comprehensive approach could also provide a greater scope for successful self-management throughout the unpredictable health trajectories of NCDs, which are relatively unique to each individual [122]. This hypothesis is in line with primary care digital health frameworks that dynamically adapt services to the clinical care pathway of the individual, which cannot be predetermined and changes regularly [123]. This approach supports our hypothesis that overly specific digital health interventions may not be the most appropriate solutions to comprehensive self-management of NCDs. A digital platform could accommodate not only the stage of NCD but also the current health state of the person living with that NCD, by providing the self-management tools required to suit individual circumstances. It would create an opportunity for users to experiment and *tinker* with evidence-based interventions [124], and experience successes and failures on their path to successful self-management, leading to new insights and skills in self-care. The evaluation of a digital platform should extend further than simply adhering to the discrete digital interventions hosted within the platform and should explore how they are used and actively incorporated into everyday life [104]. Longer evaluation trial periods may better facilitate these dynamic user health trajectories to accommodate periods of exploration and changing mindsets.

### Support and Guidance

The digital platform-like interventions included in this review had good adherence for up to 9 months, which could be attributed to the freedom for users to explore an array of digital features and identify the most relevant components for them as an individual. However, given that a digital platform would provide access to an extensive choice of discrete digital health interventions, it is important to note that the intention is not to overburden users [125]. It is possible that free navigation through a platform with various features to negotiate could be a cumbersome process [110].

Research indicates that using all available components is not necessarily more effective [56], and presenting a platform not matched to user needs could be detrimental to the overall success of the platform [126]. Thus, evidence suggests that the addition of human support in digital health can enhance user engagement, as users value reassurance and expert knowledge to guide their decision making [66,127,128]. Human support is resource intensive and can increase the overall cost and burden on health care systems, which may not be a feasible solution. Therefore, it is important to establish the extent to which further value can be added to a multicomponent digital health intervention by supplementing it with human support [127].

In view of this, our review has recognized the potential of applying existing theories and knowledge to create efficient, cost-effective automated guidance for users to make informed choices about their engagement. The digitally supported guidance offered to participants within the 7 interventions provided them with a supported pathway to choose behavior change components. This is in line with behavioral research that indicates that actively engaging participants in decision-making pathways for care can improve health outcomes [129] through factors such as improved autonomy [37], empowerment, and mastery [130]. These factors are critical in supporting people to self-manage their disease and in promoting a more *digitally engaged* patient [104].

One reason that people value human support is a sense of accountability [127]. However, this review showed that a sense of accountability was successfully acquired by several participants using automated platform-like interventions. Providing clear associations between a digital platform and *expert* health care professional advice may be enough to satisfy the desire for accountability. This is reinforced by research that suggests a key factor in promoting engagement with digital health interventions is clear endorsement by respected clinicians or *expert* organizations [110,127]. A digital platform would seek to use existing, evidence-based digital health interventions that could be put through a rigorous selection process before inclusion, driven by an extensive list of key stakeholders (eg, health care professionals, potential end users, and family members) to further endorse the trustworthiness of the platform [110].

The comprehensive development of these evidence-based, automated guiding decision pathways is another way to incorporate *expert* advice and guidance to support the trustworthiness of a digital platform. Automated eDecision support tools are currently being developed and validated to assist with meaningful adherence to interventions and health behavior by providing individualized, real-time assistance [81,131]; however, no validated tools have been described in the included literature to support navigation through complex digital platforms. This may have an impact on the usability or trustworthiness of the included digital platform-like interventions. The variation in guidance systems presented in this review points to a requirement for more research on an operative level of support to optimize engagement and create a user-centered experience for the individual.

In summary, the adaptive nature of a digital platform accommodates the requirement for flexibility in self-management support, which could facilitate a diverse range of users and life circumstances [66,132]. Longitudinal and detailed evaluations of digital platforms must be carried out to influence the lifetime of positive health-related choices and behaviors. It is important not only to evaluate the longevity of participating in such an intervention but also to explore the diverse patterns of engagement by a wide range of users.

### Limitations

This review was prospectively registered, used comprehensive search strategies across multiple databases, and reported according to PRISMA. Searches and publication of results have been conducted in an up-to-date and timely fashion. The inclusive nature of this review accommodated a broad range of NCDs, which enhances the generalizability of the findings among a wider population.

This review was not without limitations. The novel concept of a digital platform was introduced for the first time, which made it difficult to identify relevant literature. Having broadened the inclusion criterion, discrepancies in terminologies could still have led to key studies being omitted from this literature search or the misinterpretation of intervention content. Current research is focused on the development of a comprehensive eHealth taxonomy, but this is not yet wide ranging enough [133]. In response to this, a systematic approach and extensive use of terminology in search strategies were implemented to ensure that pertinent literature was included for review.

Another limitation was that none of the interventions included in this review were readily available on the web to the reviewer, none provided a digital preservation URL, and intervention descriptions were not always sufficient to identify key features. There has been a call for improved reporting of digital health interventions to improve examination and evaluation of intervention characteristics. This limitation is an important reminder that future developments should use reputable reporting frameworks and guidelines to outline their work [64,134].

### Conclusions

We have identified a gap in the research on comprehensive and flexible digital health for the self-management of NCDs. Thus, we proposed the contemporary concept of a digital platform, which supports the innovative use of already existing digital interventions for health behavior change. Initial searches indicated that no such digital platforms currently exist, which may indicate a missed opportunity to optimize user engagement with already developed, evidence-based digital interventions. In response, this review focused on digital platform-like interventions to provide an understanding of the development and contextual considerations required to optimally construct a digital platform.

Iterative, user-centered development may be associated with improved adherence and sustained use. Offering a palette of evidence-based interventions with an appropriate degree of guidance has the potential to facilitate disease-specific health behavior change and effective self-management among a myriad of users, conditions, or stages of care. This review and the novel concept of digital platform-like interventions contribute new knowledge to the rhetoric of digital health for the self-management of NCDs.

## Appendix

Multimedia Appendix 1 Search strategy for EbscoHost.

Multimedia Appendix 2 Star Rating Quality scoring of included studies. Mixed Methods Appraisal Tool (MMAT)-Version 2018.

Multimedia Appendix 3 Overview of summative evaluations.

## Abbreviations

CONSORT-EHEALTH: Consolidated Standards of Reporting Trials of Electronic and Mobile Health Applications and Online Telehealth
COPD: chronic obstructive pulmonary disease
CVD: cardiovascular disease
HAPA: Health Action Process Approach
HbA_1c_: glycated hemoglobin
MET: metabolic equivalents of task-minutes
MMAT: Mixed Methods Appraisal Tool
MVPA: moderate-to-vigorous physical activity
NCD: noncommunicable disease
PRISMA: Preferred Reporting Items for Systematic Reviews and Meta-Analyses
SCT: social cognitive theory
TTM: transtheoretical model

## References

[1] GBD 2016 Causes of Death Collaborators (2017). Global, regional, and national age-sex specific mortality for 264 causes of death, 1980-2016: a systematic analysis for the Global Burden of Disease Study 2016. Lancet.

[2] GBD 2016 DALYs HALE Collaborators (2017). Global, regional, and national disability-adjusted life-years (DALYs) for 333 diseases and injuries and healthy life expectancy (HALE) for 195 countries and territories, 1990-2016: a systematic analysis for the Global Burden of Disease Study 2016. Lancet.

[3] GBD 2016 Risk Factors Collaborators (2017). Global, regional, and national comparative risk assessment of 84 behavioural, environmental and occupational, and metabolic risks or clusters of risks, 1990-2016: a systematic analysis for the Global Burden of Disease Study 2016. Lancet.

[4] Piepoli MF, Hoes AW, Agewall S, Albus C, Brotons C, Catapano AL, Cooney M, Corrà U, Cosyns B, Deaton C, Graham I, Hall MS, Hobbs FDR, Løchen M, Löllgen H, Marques-Vidal P, Perk J, Prescott E, Redon J, Richter DJ, Sattar N, Smulders Y, Tiberi M, van DWHB, van DI, Verschuren WMM, Binno S, ESC Scientific Document Group (2016). Eur Heart J.

[5] Barquera S, Pedroza-Tobías A, Medina C, Hernández-Barrera L, Bibbins-Domingo K, Lozano R, Moran AE (2015). Global Overview of the Epidemiology of Atherosclerotic Cardiovascular Disease. Arch Med Res.

[6] Yusuf S, Hawken S, Ounpuu S, Dans T, Avezum A, Lanas F, McQueen M, Budaj A, Pais P, Varigos J, Lisheng L, INTERHEART Study Investigators (2004). Effect of potentially modifiable risk factors associated with myocardial infarction in 52 countries (the INTERHEART study): case-control study. Lancet.

[7] Ezzati M, Vander Hoorn S, Lawes CMM, Leach R, James WPT, Lopez AD, Rodgers A, Murray CJL (2005). Rethinking the “Diseases of Affluence” Paradigm: Global Patterns of Nutritional Risks in Relation to Economic Development. PLoS Med.

[8] Rich MW, Beckham V, Wittenberg C, Leven CL, Freedland KE, Carney RM (1995). A multidisciplinary intervention to prevent the readmission of elderly patients with congestive heart failure. N Engl J Med.

[9] Dalal HM, Doherty P, Taylor RS (2015). Cardiac rehabilitation. BMJ.

[10] Gardetto NJ (2011). Self-management in heart failure: where have we been and where should we go?. J Multidiscip Healthc.

[11] Norris SL, Engelgau MM, Narayan KM (2001). Effectiveness of self-management training in type 2 diabetes: a systematic review of randomized controlled trials. Diabetes Care.

[12] Zwerink M, Brusse-Keizer M, van der Valk Paul D L P M, Zielhuis GA, Monninkhof EM, van der Palen Job, Frith PA, Effing T (2014). Self management for patients with chronic obstructive pulmonary disease. Cochrane Database Syst Rev.

[13] Piepoli MF, Corrà Ugo, Adamopoulos S, Benzer W, Bjarnason-Wehrens B, Cupples M, Dendale P, Doherty P, Gaita D, Höfer Stefan, McGee H, Mendes M, Niebauer J, Pogosova N, Garcia-Porrero E, Rauch B, Schmid JP, Giannuzzi P (2014). Eur J Prev Cardiol.

[14] Bandura A (2004). Health promotion by social cognitive means. Health Educ Behav.

[15] Buckley J, Doherty P, Furze G, Jones J, Hinton S, Hayward J (2017). Standards and core components for cardiovascular disease prevention and rehabilitation. https://www.bacpr.com/resources/BACPR_Standards_and_Core_Components_2017.pdf.

[16] Woodruffe S, Neubeck L, Clark RA, Gray K, Ferry C, Finan J, Sanderson S, Briffa TG (2015). Australian Cardiovascular Health and Rehabilitation Association (ACRA) core components of cardiovascular disease secondary prevention and cardiac rehabilitation 2014. Heart Lung Circ.

[17] Spruit MA, Singh SJ, Garvey C, ZuWallack R, Nici L, Rochester C, Hill K, Holland AE, Lareau SC, Man WD, Pitta F, Sewell L, Raskin J, Bourbeau J, Crouch R, Franssen FME, Casaburi R, Vercoulen JH, Vogiatzis I, Gosselink R, Clini EM, Effing TW, Maltais F, van der Palen Job, Troosters T, Janssen DJA, Collins E, Garcia-Aymerich J, Brooks D, Fahy BF, Puhan MA, Hoogendoorn M, Garrod R, Schols AMWJ, Carlin B, Benzo R, Meek P, Morgan M, Rutten-van Mölken Maureen P M H, Ries AL, Make B, Goldstein RS, Dowson CA, Brozek JL, Donner CF, Wouters EFM, ATS/ERS Task Force on Pulmonary Rehabilitation (2013). An official American Thoracic Society/European Respiratory Society statement: key concepts and advances in pulmonary rehabilitation. Am J Respir Crit Care Med.

[18] Powers MA, Bardsley J, Cypress M, Duker P, Funnell MM, Fischl AH, Maryniuk MD, Siminerio L, Vivian E (2017). Diabetes Self-management Education and Support in Type 2 Diabetes: A Joint Position Statement of the American Diabetes Association, the American Association of Diabetes Educators, and the Academy of Nutrition and Dietetics. Diabetes Educ.

[19] Roglic Gojka, World Health Organization (2016). Global Report on Diabetes. https://www.who.int/diabetes/global-report/en/.

[20] Funnell MM, Brown TL, Childs BP, Haas LB, Hosey GM, Jensen B, Maryniuk M, Peyrot M, Piette JD, Reader D, Siminerio LM, Weinger K, Weiss MA (2009). National standards for diabetes self-management education. Diabetes Care.

[21] Inzucchi SE, Bergenstal RM, Buse JB, Diamant M, Ferrannini E, Nauck M, Peters AL, Tsapas A, Wender R, Matthews DR (2015). Management of hyperglycemia in type 2 diabetes, 2015: a patient-centered approach: update to a position statement of the American Diabetes Association and the European Association for the Study of Diabetes. Diabetes Care.

[22] Anderson L, Oldridge N, Thompson DR, Zwisler A, Rees K, Martin N, Taylor RS (2016). Exercise-Based Cardiac Rehabilitation for Coronary Heart Disease: Cochrane Systematic Review and Meta-Analysis. J Am Coll Cardiol.

[23] Deakin T, McShane CE, Cade JE, Williams RDRR (2005). Group based training for self-management strategies in people with type 2 diabetes mellitus. Cochrane Database Syst Rev.

[24] Jordan RE, Majothi S, Heneghan NR, Blissett DB, Riley RD, Sitch AJ, Price MJ, Bates EJ, Turner AM, Bayliss S, Moore D, Singh S, Adab P, Fitzmaurice DA, Jowett S, Jolly K (2015). Supported self-management for patients with moderate to severe chronic obstructive pulmonary disease (COPD): an evidence synthesis and economic analysis. Health Technol Assess.

[25] Bjarnason-Wehrens B, McGee H, Zwisler A, Piepoli MF, Benzer W, Schmid J, Dendale P, Pogosova NV, Zdrenghea D, Niebauer J, Mendes M (2010). Cardiac rehabilitation in Europe: results from the European Cardiac Rehabilitation Inventory Survey. Eur J Cardiovasc Prev Rehabil.

[26] Dunlay SM, Pack QR, Thomas RJ, Killian JM, Roger VL (2014). Participation in cardiac rehabilitation, readmissions, and death after acute myocardial infarction. Am J Med.

[27] Horigan G, Davies M, Findlay-White F, Chaney D, Coates V (2017). Reasons why patients referred to diabetes education programmes choose not to attend: a systematic review. Diabet Med.

[28] Doherty PJ, Harrison AS (2017). The National Audit of Cardiac Rehabilitation: Annual Statistical Report 2017. British Heart Foundation.

[29] Rochester CL, Vogiatzis I, Holland AE, Lareau SC, Marciniuk DD, Puhan MA, Spruit MA, Masefield S, Casaburi R, Clini EM, Crouch R, Garcia-Aymerich J, Garvey C, Goldstein RS, Hill K, Morgan M, Nici L, Pitta F, Ries AL, Singh SJ, Troosters T, Wijkstra PJ, Yawn BP, ZuWallack RL (2015). An Official American Thoracic Society/European Respiratory Society Policy Statement: Enhancing Implementation, Use, and Delivery of Pulmonary Rehabilitation. Am J Respir Crit Care Med.

[30] Ades PA, Keteyian SJ, Wright JS, Hamm LF, Lui K, Newlin K, Shepard DS, Thomas RJ (2017). Increasing Cardiac Rehabilitation Participation From 20% to 70%: A Road Map From the Million Hearts Cardiac Rehabilitation Collaborative. Mayo Clin Proc.

[31] Turk-Adawi K, Sarrafzadegan N, Grace SL (2014). Global availability of cardiac rehabilitation. Nat Rev Cardiol.

[32] Neubeck L, Freedman SB, Clark AM, Briffa T, Bauman A, Redfern J (2012). Participating in cardiac rehabilitation: a systematic review and meta-synthesis of qualitative data. Eur J Prev Cardiol.

[33] Davies P, Taylor F, Beswick A, Wise F, Moxham T, Rees K, Ebrahim S (2010). Promoting patient uptake and adherence in cardiac rehabilitation. Cochrane Database Syst Rev.

[34] Suaya JA, Shepard DS, Normand ST, Ades PA, Prottas J, Stason WB (2007). Use of cardiac rehabilitation by Medicare beneficiaries after myocardial infarction or coronary bypass surgery. Circulation.

[35] Clark AM, King-Shier KM, Thompson DR, Spaling MA, Duncan AS, Stone JA, Jaglal SB, Angus JE (2012). A qualitative systematic review of influences on attendance at cardiac rehabilitation programs after referral. Am Heart J.

[36] Harrison AS, Doherty P, Phillips A (2018). An analysis of barriers to entry of cardiac rehabilitation in patients with diabetes: Using data from the National Audit of Cardiac Rehabilitation. Diab Vasc Dis Res.

[37] Clark RA, Conway A, Poulsen V, Keech W, Tirimacco R, Tideman P (2015). Alternative models of cardiac rehabilitation: a systematic review. Eur J Prev Cardiol.

[38] Pal K, Dack C, Ross J, Michie S, May C, Stevenson F, Farmer A, Yardley L, Barnard M, Murray E (2018). Digital Health Interventions for Adults With Type 2 Diabetes: Qualitative Study of Patient Perspectives on Diabetes Self-Management Education and Support. J Med Internet Res.

[39] Lahham A, McDonald CF, Mahal A, Lee AL, Hill CJ, Burge AT, Cox NS, Moore R, Nicolson C, O'Halloran P, Gillies R, Holland AE (2018). Home-based pulmonary rehabilitation for people with COPD: A qualitative study reporting the patient perspective. Chron Respir Dis.

[40] Holland AE, Mahal A, Hill CJ, Lee AL, Burge AT, Cox NS, Moore R, Nicolson C, O'Halloran P, Lahham A, Gillies R, McDonald CF (2017). Home-based rehabilitation for COPD using minimal resources: a randomised, controlled equivalence trial. Thorax.

[41] Anderson L, Sharp GA, Norton RJ, Dalal H, Dean SG, Jolly K, Cowie A, Zawada A, Taylor RS (2017). Home-based versus centre-based cardiac rehabilitation. Cochrane Database Syst Rev.

[42] Rawstorn JC, Gant N, Direito A, Beckmann C, Maddison R (2016). Telehealth exercise-based cardiac rehabilitation: a systematic review and meta-analysis. Heart.

[43] NCES Global digital population as of April 2019 (in millions). Statista:The Statistics Portal.

[44] NCES Number of smartphone users worldwide from 2014 to 2020 (in billions). Statista:The Statistics Portal.

[45] NCES (2019). Statista:The Statistics Portal.

[46] Webb TL, Joseph J, Yardley L, Michie S (2010). Using the internet to promote health behavior change: a systematic review and meta-analysis of the impact of theoretical basis, use of behavior change techniques, and mode of delivery on efficacy. J Med Internet Res.

[47] Maddison R, Rawstorn JC, Shariful Islam SM, Ball K, Tighe S, Gant N, Whittaker RM, Chow CK (2019). mHealth Interventions for Exercise and Risk Factor Modification in Cardiovascular Disease. Exerc Sport Sci Rev.

[48] Hanlon P, Daines L, Campbell C, McKinstry B, Weller D, Pinnock H (2017). Telehealth Interventions to Support Self-Management of Long-Term Conditions: A Systematic Metareview of Diabetes, Heart Failure, Asthma, Chronic Obstructive Pulmonary Disease, and Cancer. J Med Internet Res.

[49] Pfaeffli Dale L, Whittaker R, Jiang Y, Stewart R, Rolleston A, Maddison R (2015). Text Message and Internet Support for Coronary Heart Disease Self-Management: Results From the Text4Heart Randomized Controlled Trial. J Med Internet Res.

[50] Dobson R, Whittaker R, Jiang Y, Maddison R, Shepherd M, McNamara C, Cutfield R, Khanolkar M, Murphy R (2018). Effectiveness of text message based, diabetes self management support programme (SMS4BG): two arm, parallel randomised controlled trial. BMJ.

[51] Chow CK, Redfern J, Hillis GS, Thakkar J, Santo K, Hackett ML, Jan S, Graves N, de KL, Barry T, Bompoint S, Stepien S, Whittaker R, Rodgers A, Thiagalingam A (2015). Effect of Lifestyle-Focused Text Messaging on Risk Factor Modification in Patients With Coronary Heart Disease: A Randomized Clinical Trial. JAMA.

[52] Pfaeffli Dale L, Dobson R, Whittaker R, Maddison R (2016). The effectiveness of mobile-health behaviour change interventions for cardiovascular disease self-management: A systematic review. Eur J Prev Cardiol.

[53] Kitsiou S, Paré G, Jaana M, Gerber B (2017). Effectiveness of mHealth interventions for patients with diabetes: An overview of systematic reviews. PLoS One.

[54] Maddison R, Rawstorn JC, Stewart RAH, Benatar J, Whittaker R, Rolleston A, Jiang Y, Gao L, Moodie M, Warren I, Meads A, Gant N (2018). Effects and costs of real-time cardiac telerehabilitation: randomised controlled non-inferiority trial. Heart.

[55] Varnfield M, Karunanithi M, Lee C, Honeyman E, Arnold D, Ding H, Smith C, Walters DL (2014). Smartphone-based home care model improved use of cardiac rehabilitation in postmyocardial infarction patients: results from a randomised controlled trial. Heart.

[56] Eysenbach G (2005). The law of attrition. J Med Internet Res.

[57] World Health Organisation (2016). Monitoring and evaluating digital health interventions: a practical guide to conducting research and assessment. https://apps.who.int/iris/bitstream/handle/10665/252183/9789241511766-eng.pdf.

[58] Stellefson M, Chaney B, Barry AE, Chavarria E, Tennant B, Walsh-Childers K, Sriram PS, Zagora J (2013). Web 2.0 chronic disease self-management for older adults: a systematic review. J Med Internet Res.

[59] Kuijpers W, Groen WG, Aaronson NK, van Harten WH (2013). A systematic review of web-based interventions for patient empowerment and physical activity in chronic diseases: relevance for cancer survivors. J Med Internet Res.

[60] Richardson WS, Wilson MC, Nishikawa J, Hayward RS (1995). The well-built clinical question: a key to evidence-based decisions. ACP J Club.

[61] Davies KS (2011). Formulating the Evidence Based Practice Question: A Review of the Frameworks. Evidence Based Library and Information Practice.

[62] Moher D, Liberati A, Tetzlaff J, Altman DG (2009). Preferred reporting items for systematic reviews and meta-analyses: the PRISMA statement. Ann Intern Med.

[63] Hong Qn, Fàbregues S, Bartlett G, Boardman F, Cargo M, Dagenais P, Gagnon M, Griffiths F, Nicolau B, O’Cathain A, Rousseau M, Vedel I, Pluye P (2018). The Mixed Methods Appraisal Tool (MMAT) version 2018 for information professionals and researchers. EFI.

[64] Eysenbach G, CONSORT EHEALTH GROUP (2011). CONSORT-EHEALTH: improving and standardizing evaluation reports of Web-based and mobile health interventions. J Med Internet Res.

[65] Craig P, Dieppe P, Macintyre S, Michie S, Nazareth I, Petticrew M (2013). Developing and evaluating complex interventions: the new Medical Research Council guidance. Int J Nurs Stud.

[66] Michie S, Yardley L, West R, Patrick K, Greaves F (2017). Developing and Evaluating Digital Interventions to Promote Behavior Change in Health and Health Care: Recommendations Resulting From an International Workshop. J Med Internet Res.

[67] Whittaker R, Merry S, Dorey E, Maddison R (2012). A development and evaluation process for mHealth interventions: examples from New Zealand. J Health Commun.

[68] Sieverink F, Kelders SM, van Gemert-Pijnen JE (2017). Clarifying the Concept of Adherence to eHealth Technology: Systematic Review on When Usage Becomes Adherence. J Med Internet Res.

[69] Antypas K, Wangberg SC (2012). E-Rehabilitation - an Internet and mobile phone based tailored intervention to enhance self-management of cardiovascular disease: study protocol for a randomized controlled trial. BMC Cardiovasc Disord.

[70] Antypas K, Wangberg SC (2014). Combining users' needs with health behavior models in designing an internet- and mobile-based intervention for physical activity in cardiac rehabilitation. JMIR Res Protoc.

[71] Antypas K, Wangberg SC (2014). An Internet- and mobile-based tailored intervention to enhance maintenance of physical activity after cardiac rehabilitation: short-term results of a randomized controlled trial. J Med Internet Res.

[72] Poppe L, Van der Mispel C, De Bourdeaudhuij I, Verloigne M, Shadid S, Crombez G (2017). Users' thoughts and opinions about a self-regulation-based eHealth intervention targeting physical activity and the intake of fruit and vegetables: A qualitative study. PLoS One.

[73] Poppe L, Crombez G, De Bourdeaudhuij I, Van der Mispel C, Shadid S, Verloigne M (2018). Experiences and Opinions of Adults with Type 2 Diabetes Regarding a Self-Regulation-Based eHealth Intervention Targeting Physical Activity and Sedentary Behaviour. Int J Environ Res Public Health.

[74] Voncken-Brewster V, Tange H, de Vries H, Nagykaldi Z, Winkens B, van der Weijden T (2013). A randomised controlled trial testing a web-based, computer-tailored self-management intervention for people with or at risk for chronic obstructive pulmonary disease: a study protocol. BMC Public Health.

[75] Voncken-Brewster V, Moser A, van der Weijden T, Nagykaldi Z, de Vries H, Tange H (2013). Usability evaluation of an online, tailored self-management intervention for chronic obstructive pulmonary disease patients incorporating behavior change techniques. JMIR Res Protoc.

[76] Voncken-Brewster V, Tange H, Moser A, Nagykaldi Z, de Vries H, van der Weijden T (2014). Integrating a tailored e-health self-management application for chronic obstructive pulmonary disease patients into primary care: a pilot study. BMC Fam Pract.

[77] Voncken-Brewster V, Tange H, de Vries H, Nagykaldi Z, Winkens B, van der Weijden T (2015). A randomized controlled trial evaluating the effectiveness of a web-based, computer-tailored self-management intervention for people with or at risk for COPD. Int J Chron Obstruct Pulmon Dis.

[78] Voncken-Brewster V, Amoureus M, de Vries H, Nagykaldi Z, Winkens B, van der Weijden T, Tange H (2017). The Impact of Participant Characteristics on Use and Satisfaction of a Web-Based Computer-Tailored Chronic Obstructive Pulmonary Disease Self-Management Intervention: A Process Evaluation. JMIR Form Res.

[79] Claes J, Buys R, Woods C, Briggs A, Geue C, Aitken M, Moyna N, Moran K, McCaffrey N, Chouvarda I, Walsh D, Budts W, Filos D, Triantafyllidis A, Maglaveras N, Cornelissen VA (2017). PATHway I: design and rationale for the investigation of the feasibility, clinical effectiveness and cost-effectiveness of a technology-enabled cardiac rehabilitation platform. BMJ Open.

[80] Walsh DM, Moran K, Cornelissen V, Buys R, Cornelis N, Woods C (2018). Electronic Health Physical Activity Behavior Change Intervention to Self-Manage Cardiovascular Disease: Qualitative Exploration of Patient and Health Professional Requirements. J Med Internet Res.

[81] Walsh DMJ, Moran K, Cornelissen V, Buys R, Claes J, Zampognaro P, Melillo F, Maglaveras N, Chouvarda I, Triantafyllidis A, Filos D, Woods CB (2019). The development and codesign of the PATHway intervention: a theory-driven eHealth platform for the self-management of cardiovascular disease. Transl Behav Med.

[82] Claes J, Cornelissen V, McDermott C, Moyna N, Pattyn N, Cornelis N, Gallagher A, McCormack C, Newton H, Gillain A, Budts W, Goetschalckx K, Woods C, Moran K, Buys R (2020). Feasibility, Acceptability, and Clinical Effectiveness of a Technology-Enabled Cardiac Rehabilitation Platform (Physical Activity Toward Health-I): Randomized Controlled Trial. J Med Internet Res.

[83] Yu CH, Parsons J, Mamdani M, Lebovic G, Shah BR, Bhattacharyya O, Laupacis A, Straus SE (2012). Designing and evaluating a web-based self-management site for patients with type 2 diabetes--systematic website development and study protocol. BMC Med Inform Decis Mak.

[84] Yu CH, Parsons JA, Hall S, Newton D, Jovicic A, Lottridge D, Shah BR, Straus SE (2014). User-centered design of a web-based self-management site for individuals with type 2 diabetes - providing a sense of control and community. BMC Med Inform Decis Mak.

[85] Yu CH, Parsons JA, Mamdani M, Lebovic G, Hall S, Newton D, Shah BR, Bhattacharyya O, Laupacis A, Straus SE (2014). A web-based intervention to support self-management of patients with type 2 diabetes mellitus: effect on self-efficacy, self-care and diabetes distress. BMC Med Inform Decis Mak.

[86] Hofmann M, Dack C, Barker C, Murray E (2016). The Impact of an Internet-Based Self-Management Intervention (HeLP-Diabetes) on the Psychological Well-Being of Adults with Type 2 Diabetes: A Mixed-Method Cohort Study. J Diabetes Res.

[87] Alkhaldi G, Modrow K, Hamilton F, Pal K, Ross J, Murray E (2017). Promoting Engagement With a Digital Health Intervention (HeLP-Diabetes) Using Email and Text Message Prompts: Mixed-Methods Study. Interact J Med Res.

[88] Murray E, Sweeting M, Dack C, Pal K, Modrow K, Hudda M, Li J, Ross J, Alkhaldi G, Barnard M, Farmer A, Michie S, Yardley L, May C, Parrott S, Stevenson F, Knox M, Patterson D (2017). Web-based self-management support for people with type 2 diabetes (HeLP-Diabetes): randomised controlled trial in English primary care. BMJ Open.

[89] Li J, Parrott S, Sweeting M, Farmer A, Ross J, Dack C, Pal K, Yardley L, Barnard M, Hudda M, Alkhaldi G, Murray E (2018). Cost-Effectiveness of Facilitated Access to a Self-Management Website, Compared to Usual Care, for Patients With Type 2 Diabetes (HeLP-Diabetes): Randomized Controlled Trial. J Med Internet Res.

[90] Poduval S, Ahmed S, Marston L, Hamilton F, Murray E (2018). Crossing the Digital Divide in Online Self-Management Support: Analysis of Usage Data From HeLP-Diabetes. JMIR Diabetes.

[91] Dack C, Ross J, Stevenson F, Pal K, Gubert E, Michie S, Yardley L, Barnard M, May C, Farmer A, Wood B, Murray E (2019). A digital self-management intervention for adults with type 2 diabetes: Combining theory, data and participatory design to develop HeLP-Diabetes. Internet Interv.

[92] Poppe L, De Bourdeaudhuij I, Verloigne M, Degroote L, Shadid S, Crombez G (2019). A Self-Regulation-Based eHealth and mHealth Intervention for an Active Lifestyle in Adults With Type 2 Diabetes: Protocol for a Randomized Controlled Trial. JMIR Res Protoc.

[93] Poppe L, De Bourdeaudhuij I, Verloigne M, Shadid S, Van Cauwenberg J, Compernolle S, Crombez G (2019). Efficacy of a Self-Regulation-Based Electronic and Mobile Health Intervention Targeting an Active Lifestyle in Adults Having Type 2 Diabetes and in Adults Aged 50 Years or Older: Two Randomized Controlled Trials. J Med Internet Res.

[94] Sakakibara BM, Ross E, Arthur G, Brown-Ganzert L, Petrin S, Sedlak T, Lear SA (2017). Using Mobile-Health to Connect Women with Cardiovascular Disease and Improve Self-Management. Telemed J E Health.

[95] Sakakibara BM, Chakrabarti S, Krahn A, Mackay MH, Sedlak T, Singer J, Whitehurst DG, Lear SA (2019). Delivery of Peer Support Through a Self-Management mHealth Intervention (Healing Circles) in Patients With Cardiovascular Disease: Protocol for a Randomized Controlled Trial. JMIR Res Protoc.

[96] Murray E, Dack C, Barnard M, Farmer A, Li J, Michie S, Pal K, Parrott S, Ross J, Sweeting M, Wood B, Yardley L (2015). HeLP-Diabetes: randomised controlled trial protocol. BMC Health Serv Res.

[97] Michie S, Richardson M, Johnston M, Abraham C, Francis J, Hardeman W, Eccles MP, Cane J, Wood CE (2013). The behavior change technique taxonomy (v1) of 93 hierarchically clustered techniques: building an international consensus for the reporting of behavior change interventions. Ann Behav Med.

[98] Toobert DJ, Hampson SE, Glasgow RE (2000). The summary of diabetes self-care activities measure: results from 7 studies and a revised scale. Diabetes Care.

[99] Osborne RH, Elsworth GR, Whitfield K (2007). The Health Education Impact Questionnaire (heiQ): an outcomes and evaluation measure for patient education and self-management interventions for people with chronic conditions. Patient Educ Couns.

[100] van der Molen T, Willemse BWM, Schokker S, ten Hacken NH, Postma DS, Juniper EF (2003). Development, validity and responsiveness of the Clinical COPD Questionnaire. Health Qual Life Outcomes.

[101] Wilson PW, D'Agostino RB, Levy D, Belanger AM, Silbershatz H, Kannel WB (1998). Prediction of coronary heart disease using risk factor categories. Circulation.

[102] Polonsky WH, Fisher L, Earles J, Dudl RJ, Lees J, Mullan J, Jackson RA (2005). Assessing psychosocial distress in diabetes: development of the diabetes distress scale. Diabetes Care.

[103] Simonsen J, Robertson T, Simonsen J, Robertson T (2013). Participatory Design: an introduction. Routledge International Handbook of Participatory Design.

[104] Lupton D (2013). The digitally engaged patient: Self-monitoring and self-care in the digital health era. Soc Theory Health.

[105] Yardley L, Morrison L, Bradbury K, Muller I (2015). The person-based approach to intervention development: application to digital health-related behavior change interventions. J Med Internet Res.

[106] Bossen C, Dindler C, Garde J, Pipek V (2014). Evaluation, sustainability and long-term effects of participatory design projects. Proceedings of the 13th Participatory Design Conference: Short Papers, Industry Cases, Workshop Descriptions, Doctoral Consortium papers, and Keynote abstracts - Volume 2.

[107] Andersen TO, Bansler JP, Kensing F, Moll J (2017). From Prototype to Product: Making Participatory Design of mHealth Commercially Viable. Stud Health Technol Inform.

[108] Nelson LA, Coston TD, Cherrington AL, Osborn CY (2016). Patterns of User Engagement with Mobile- and Web-Delivered Self-Care Interventions for Adults with T2DM: A Review of the Literature. Curr Diab Rep.

[109] Chow CK, Ariyarathna N, Islam SMS, Thiagalingam A, Redfern J (2016). mHealth in Cardiovascular Health Care. Heart Lung Circ.

[110] O'Connor S, Hanlon P, O'Donnell CA, Garcia S, Glanville J, Mair FS (2016). Understanding factors affecting patient and public engagement and recruitment to digital health interventions: a systematic review of qualitative studies. BMC Med Inform Decis Mak.

[111] Danaher BG, McKay HG, Seeley JR (2005). The information architecture of behavior change websites. J Med Internet Res.

[112] Ritterband LM, Thorndike FP, Cox DJ, Kovatchev BP, Gonder-Frederick LA (2009). A behavior change model for internet interventions. Ann Behav Med.

[113] Michie Susan, Johnston M (2012). Theories and techniques of behaviour change: Developing a cumulative science of behaviour change. Health Psychology Review.

[114] Prochaska JJ, Prochaska JO (2011). A Review of Multiple Health Behavior Change Interventions for Primary Prevention. Am J Lifestyle Med.

[115] Drca N, Wolk A, Jensen-Urstad M, Larsson SC (2015). Physical activity is associated with a reduced risk of atrial fibrillation in middle-aged and elderly women. Heart.

[116] Trost SG, Owen N, Bauman AE, Sallis JF, Brown W (2002). Correlates of adults' participation in physical activity: review and update. Med Sci Sports Exerc.

[117] Brownson RC, Eyler AA, King AC, Brown DR, Shyu YL, Sallis JF (2000). Patterns and correlates of physical activity among US women 40 years and older. Am J Public Health.

[118] Buczkowski K, Marcinowicz L, Czachowski S, Piszczek E (2014). Motivations toward smoking cessation, reasons for relapse, and modes of quitting: results from a qualitative study among former and current smokers. Patient Prefer Adherence.

[119] Stults-Kolehmainen MA, Sinha R (2014). The effects of stress on physical activity and exercise. Sports Med.

[120] Bauman AE, Reis RS, Sallis JF, Wells JC, Loos RJF, Martin BW, Lancet Physical Activity Series Working Group (2012). Correlates of physical activity: why are some people physically active and others not?. Lancet.

[121] Torres SJ, Nowson CA (2007). Relationship between stress, eating behavior, and obesity. Nutrition.

[122] Murray SA, Kendall M, Boyd K, Sheikh A (2005). Illness trajectories and palliative care. BMJ.

[123] Phanareth K, Vingtoft S, Christensen AS, Nielsen JS, Svenstrup J, Berntsen GKR, Newman SP, Kayser L (2017). The Epital Care Model: A New Person-Centered Model of Technology-Enabled Integrated Care for People With Long Term Conditions. JMIR Res Protoc.

[124] Mol A, Moser I, Pols J (2010). Care in Practice On Tinkering in Clinics, Homes and Farms.

[125] May CR, Eton DT, Boehmer K, Gallacher K, Hunt K, MacDonald S, Mair FS, May CM, Montori VM, Richardson A, Rogers AE, Shippee N (2014). Rethinking the patient: using Burden of Treatment Theory to understand the changing dynamics of illness. BMC Health Serv Res.

[126] Prochaska JO, Velicer WF (1997). The transtheoretical model of health behavior change. Am J Health Promot.

[127] Yardley L, Spring BJ, Riper H, Morrison LG, Crane DH, Curtis K, Merchant GC, Naughton F, Blandford A (2016). Understanding and Promoting Effective Engagement With Digital Behavior Change Interventions. Am J Prev Med.

[128] Moller AC, Merchant G, Conroy DE, West R, Hekler E, Kugler KC, Michie S (2017). Applying and advancing behavior change theories and techniques in the context of a digital health revolution: proposals for more effectively realizing untapped potential. J Behav Med.

[129] Greene J, Hibbard JH (2012). Why does patient activation matter? An examination of the relationships between patient activation and health-related outcomes. J Gen Intern Med.

[130] Lorig KR, Holman H (2003). Self-management education: history, definition, outcomes, and mechanisms. Ann Behav Med.

[131] Triantafyllidis A, Filos D, Buys R, Claes J, Cornelissen V, Kouidi E, Chatzitofis A, Zarpalas D, Daras P, Walsh D, Woods C, Moran K, Maglaveras N, Chouvarda I (2018). Computerized decision support for beneficial home-based exercise rehabilitation in patients with cardiovascular disease. Comput Methods Programs Biomed.

[132] Schwarzer R (2008). Modeling Health Behavior Change: How to Predict and Modify the Adoption and Maintenance of Health Behaviors. Applied Psychology.

[133] Shaw T, McGregor D, Brunner M, Keep M, Janssen A, Barnet S (2017). What is eHealth (6)? Development of a Conceptual Model for eHealth: Qualitative Study with Key Informants. J Med Internet Res.

[134] Craig P, Dieppe P, Macintyre S, Michie S, Nazareth I, Petticrew M, Medical Research Council Guidance (2008). Developing and evaluating complex interventions: the new Medical Research Council guidance. BMJ.

