# Supplementary material for: Toward a Digital Platform for the Self-Management of Noncommunicable Disease: Systematic Review of Platform-Like Interventions
Source: J Med Internet Res. 2020 Oct 28;22(10):e16774. doi: 10.2196/16774 (PMC7657720; doi:10.2196/16774)
Supplement: Multimedia Appendix 1 [file jmir_v22i10e16774_app1.pdf]

Multimedia Appendix 1

Search Strategy for EbscoHost

1	"long term condition*" OR "long term ill*" OR "chronic disease*" OR "chronic* ill*" OR "chronic condition*" OR "non communicable" OR noncommunicable OR NCD* OR comorbid*
2	web* OR digital* OR on#line OR remote* OR internet OR tech* OR mobile*
3	platform* OR hub OR portal OR suite OR toolbox OR program* OR system* OR intervention* OR "multi component" OR multicomponent OR multimodal OR multiple
4	"health related behavio#r*" OR behavio#r* OR "self manag*" OR "secondary prevent*" OR "health behavio#r" OR "behavio#r* change*" OR "risk factor*" OR "lifestyle risk*" OR "health risk*"
5	1 AND 2 AND 3 AND 4
6	"cardi* disease*" OR cardi* OR "heart disease*" OR diabet* OR "chronic obstructive pulmonary disease" OR "chronic respir*" OR "high blood pressure" OR "hypertensi*" OR "metabolic syndrome"
7	(1 OR 6) AND 2 AND 3 AND 4
8	"web based" OR "digital health" OR "internet based" OR "tech* based" OR "tech* enabled" OR "health tech*" OR eHealth OR "electronic health" OR "mob* health*" OR "mob* app*" OR "mob* tech*" OR mHealth OR telemonitor* OR telehealth OR telemedicine OR telerehabilitat* OR "personali?ed health" OR wearable* OR "wearable tech*"
9	(1 OR 6) AND (2 OR 8) AND 3 AND 4
10	"secondary rehabilitat*" OR "disease manage*" OR rehabilitat* OR "self monitor*" OR prevent* OR "symptom monitor*" OR adherence OR "self help" OR "self care" OR "risk manag*" OR "individual* care"
11	(1 OR 6) AND (2 OR 8) AND 3 AND (4 OR 10)
12	11 AND (Published Date: 19900101-20200120; Language: English)
