# Supplementary material for: Toward a Digital Platform for the Self-Management of Noncommunicable Disease: Systematic Review of Platform-Like Interventions
Source: J Med Internet Res. 2020 Oct 28;22(10):e16774. doi: 10.2196/16774 (PMC7657720; doi:10.2196/16774)
Supplement: Multimedia Appendix 2 [file jmir_v22i10e16774_app2.pdf]

	Antypas & Wangberg [71]	Murray et al [88]	Poppe et al [93]	Sakakibara et al [94]	Voncken-Brewster et al [77]	Walsh et al [82]	Yu et al [85]
**SCREENING QUESTIONS**							
S1. Are there clear research questions?	Yes	Yes	Yes	Yes	Yes	Yes	Yes
S2. Do the collected data allow to address the research questions?	Yes	Yes	Yes	Yes	Yes	Yes	Yes
**1. QUALITATIVE STUDIES**	-	-	-	-	-	-	-
**2. RANDOMIZED CONTROLLED TRIALS**							
2.1. Is randomization appropriately performed?	Yes	Yes	Yes		Yes	Yes^	
2.2. Are the groups comparable at baseline?	Can't Tell^	Yes	Yes		Yes	Yes	
2.3. Are there complete outcome data?	Can't Tell^	No	No		No	No	
2.4. Are outcome assessors blinded to the intervention provided?	No	Yes	No		Yes	Yes	
2.5 Did the participants adhere to the assigned intervention?	No	No	No		No	No	
**3. NON-RANDOMIZED STUDIES**	-	-	-	-	-	-	-
**4. QUANTITATIVE DESCRIPTIVE STUDIES**	-	-	-	-	-	-	-
**5. MIXED METHODS STUDIES**							
5.1. Is there an adequate rationale for using a mixed methods design to address the research question?				Yes			Yes
5.2. Are the different components of the study effectively integrated to answer the research question?				Yes			Yes
5.3. Are the outputs of the integration of qualitative and quantitative components adequately interpreted?				No			Yes
5.4. Are divergences and inconsistencies between quantitative and qualitative results adequately addressed?				Yes			Yes
5.5. Do the different components of the study adhere to the quality criteria of each tradition of the methods involved?				No			Yes
**Total Score (Yes=1, No=0) Range 0-7**	**3**	**5**	**4**	**5**	**5**	**5**	**7**
**%**	43	71	57	71	71	71	100
**Star Rating**	**	***	***	***	***	***	****

^referred to companion paper for clarification
