# Supplementary material for: Toward a Digital Platform for the Self-Management of Noncommunicable Disease: Systematic Review of Platform-Like Interventions
Source: J Med Internet Res. 2020 Oct 28;22(10):e16774. doi: 10.2196/16774 (PMC7657720; doi:10.2196/16774)
Supplement: Multimedia Appendix 3 [file jmir_v22i10e16774_app3.pdf]

	Study Design	Target Group (No. Participants, Mean Age)	Intervention Length (data collection)	Outcome Categories	Main Results
Antypas & Wangberg [71]	RCT[a]	n=69, 59.5 years	3-months (at 1 & 3-months)	Behavioral, Psychosocial	**3 months:** IG[b] significantly higher overall PA[d] (5613 MET[e]-mins/week, IQR 2828) than CG[c] (1356 MET[e]-min/week, IQR 2937)(*P*=.02). Significance reported for walking (+453.80 MET[e]-mins/week, *P*<.05) not for moderate/vigorous activity.
Murray et al [88]	RCT[a]	n=374, 65 years	12-months (at 3 & 12-months)	Psychosocial, Clinical	**12 months:** significant difference in HbA1c. IG lower HbA1c than CG (mean difference= -0.24%; 95% CIs −0.44 to −0.049, *P*=.014). Causal analyses indicate 'high-usage' for 12 months could average reduction in HbA1c by -0.44% (95% CI −0.81 to −0.06).
Poppe et al [93]	RCT[a]	n=54, 62.7 years	5-weeks (at 5-weeks)	Behavioral, Psychosocial	**5 weeks:** significant intervention effect favoring PA IG was found for accelerometer-assessed MVPA (effect size= 0.84,*P*=.049). Time effect for PA IG baseline (Mean 17.1 mins/day (SD 15.7)) to 5-weeks (Mean 25.5 mins/day (SD 15.8)) A significant IG effect favoring SB IG was found for accelerometer-assessed daily breaks from sedentary time (*P*=.005). Significant time*group interaction

					effect favoring IG found for self-monitoring (effect size=0.54, *P*=.008).
Sakakibara et al [94]	Pre-Post	n=35, 57 years (median)	10-weeks (at 10-weeks)	Behavioral, Psychosocial	**10 weeks:** significant improvements in three self-management domains: health behaviors (z=-2.11, *P*=.04), self-monitoring (z=-2.04, *P*=.04) and social support (z=-2.58, *P*=.01) domains. **10 weeks:** significant improvements in social integration (z=-3.01, *P*=.002) i.e. domain of social support survey.
Voncken-Brewster et al [77]	RCT[a]	n= 1325, 57.6 years	6-months (at 6-months)	Behavioral, Psychosocial, Clinical	**6 months:** Significant improvement (uncorrected effect) in clinical disease control (b=-.06, 95% CI -0.11 to -0.01, *P*=.010)
Walsh et al [82]	RCT[a]	n= 120, 61.4 years	6-months (at 3 & 6-months)	Behavioral, Psychosocial, Clinical	**6 months:** significant MVPA increase for IG (*P*=.01), from baseline (Mean 127 mins/day (SD 57.9)) to 6-months (Mean 141 mins/day (SD 69.1)). Significant interaction effect between IG v CG over time (effect size= 0.42, P=.04). **6 months:** Significant group interaction in favour of IG for cardiovascular risk score (effect size=-0.36 , *P*=.03). Significant group interaction in favour of IG for diastolic blood pressure (effect size= -0.49, *P*=.004).

Yu et al [85]	Pre-Post	n=81, 57.2 years	9-months (Self-Efficacy: every 3-weeks, Clinical: at 3, 6, 9-months)	Behavioral, Psychosocial, Clinical	**9-months:** Within IG[b], significant improvements in self-care (0.44, 95% CI 0.23-0.63)(*P*<.001), positively correlated with age (0.04/year, 95% CI 0.02-0.06)(*P*<.001) **9-months:** Within IG[b], compared intervention users and non-users for diabetes-specific QOL[f] (-4.7 v -0.9)(*P*<.001).

[a]randomized control trial. [b]intervention group. [c]control group. [d]physical activity. [e]metabolic equivalent of task. [f]quality of life
